# Brassinosteroids (BRs) Role in Plant Development and Coping with Different Stresses

**DOI:** 10.3390/ijms23031012

**Published:** 2022-01-18

**Authors:** Hakim Manghwar, Amjad Hussain, Qurban Ali, Fen Liu

**Affiliations:** 1 Lushan Botanical Garden, Chinese Academy of Sciences, Jiujiang 332000, China; 2 National Key Laboratory of Crop Genetic Improvement, Huazhong Agricultural University, Wuhan 430070, China; ad_hn@outlook.com; 3 Key Laboratory of Monitoring and Management of Crop Diseases and Pest Insects, Ministry of Education, Nanjing Agricultural University, Nanjing 210095, China; qurbanalirattar@webmail.hzau.edu.cn

**Keywords:** brassinosteroids (BRs), plant, biotic stress, abiotic stress

## Abstract

Plants are vulnerable to a number of abiotic and biotic stresses that cause a substantial decrease in the production of plants. Plants respond to different environmental stresses by experiencing a series of molecular and physiological changes coordinated by various phytohormones. The use of phytohormones to alleviate stresses has recently achieved increasing interest. Brassinosteroids (BRs) are a group of polyhydroxylated steroidal phytohormones that are required for the development, growth, and productivity of plants. These hormones are involved in regulating the division, elongation, and differentiation of numerous cell types throughout the entire plant life cycle. BR studies have drawn the interest of plant scientists over the last few decades due to their flexible ability to mitigate different environmental stresses. BRs have been shown in numerous studies to have a positive impact on plant responses to various biotic and abiotic stresses. BR receptors detect the BR at the cell surface, triggering a series of phosphorylation events that activate the central transcription factor (TF) Brassinazole-resistant 1 (BZR1), which regulates the transcription of BR-responsive genes in the nucleus. This review discusses the discovery, occurrence, and chemical structure of BRs in plants. Furthermore, their role in the growth and development of plants, and against various stresses, is discussed. Finally, BR signaling in plants is discussed.

## 1. Introduction

Plants are exposed to a wide range of biotic and abiotic stresses throughout their life cycle and need to constantly regulate their physiological and developmental processes for responding to numerous internal and external stimuli [1]. Various biotic and abiotic stresses significantly contribute to major global crop production losses by primarily influencing the stress tolerance/adaptive ability of plants [2]. Plants utilize various signaling molecules, including hormones for mediating the plant response to the number of stresses [3,4]. Phytohormones have been widely considered as the natural activators for plant growth and development. They maintain healthy life in plants, and play an essential role in defense mechanisms against various stresses [5]. Phytohormones initiate a signaling cascade that involves a number of molecular players, which lead to an ideal generic pathway [6]. Brassinosteroids (BRs) are an important group of plant hormones involved in regulating plant growth and development, and they help plants to adapt to the environment [7]. Biosynthesis and signaling of the hormone have been extensively studied since its discovery, particularly in the Arabidopsis (*Arabidopsis thaliana*), which led to a comprehensive understanding of BR synthesis and its signaling pathways [8,9]. Here, we discuss the discovery, occurrence, and the chemical structure of BRs in plants. Moreover, the role of BRs in plant growth and development, and against various kinds of stresses, is discussed. Finally, their signaling in plants is discussed.

## 2. Discovery of BRs in Different Plant Species

BRs were initially discovered in *Brassica napus* pollen on the basis of their ability to promote growth [10]. BRs have been discovered as stimulants for plant cell elongation and division. BRs were subsequently named ‘brassins’. Brassinolide (BL), the most active BR, was isolated in 1979 [11]. The most significant finding was the isolation of Brassinosteroid insensitive 1 (BRI1)—a receptor kinase that triggers an intracellular signaling cascade in response to extracellular BR perception [12]. Since the discovery of BL, a huge number of chemically different BRs have been discovered throughout the plant kingdom, including green algae and land plants, suggesting that BRs evolved early during plant evolution. BRs were identified as plant hormones after discovering BR-deficient mutants in *A. thaliana* [13]. Among all BRs studied to date, Castasterone (CS), Typhasterol (TY), Brassinolide (BL), 6-deoxocastasterone (6-deoxoCS), 28-norcastasterone (28-norCS), and Teasterone (TE) are commonly present in various plant species throughout different environments [14,15,16].

## 3. Occurrence of BRs in Plants

BRs have been found in organs and all parts of the plants, such as leaves, stems, roots, flowers, pollen, anthers, and seeds [17,18]. BR is universally distributed in all growing tissues of higher plants, but significantly higher concentrations have been detected in seed, pollen, and fruit [19]. The level of BR in the young tissues (1–100 ng/g fresh weight) is normally higher than in mature ones (0.01–0.1 ng/g fresh weight) [20]. BL and CS are the most significant BRs because of their higher biological activity and widespread distribution in plants. However, due to its commercial availability, 24-epibrassinolide (EBR) is the most commonly used BR for studying the physiological effects of exogenous steroid phytohormones on plants [21].

After the BL discovery, about 69 BRs have been identified in 64 plant species, including 6 gymnosperms, 53 angiosperms (41 dicotyledons and 12 monocotyledons), 1 pteridophyte (*Equisetum arvense*), 3 algae (*Hydrodictyon reticulatum, Cystoseira myrica,* and *Chlorella Vulgaris*) and 1 bryophyte (*Marchantia polymorpha*) [17,18], a moss (*Physcomitrella patens*), lycophytes (*Selaginella moellendorffii* and *S. uncinata*), and 13 fern species [22]. The concentration of 6-deoxotyphasterol (6-deoxoTY) concentration was found to be 6400-fold greater than BL in the pollen of *Cupressus arizonica*. Additionally, the highest concentration of BR, 6.4 mg 6-deoxoTY per kilogram (kg) pollen, has been found in *C. arizonica* [17]. Only 52 BRs have been reported in terms of their biological activities in plants [16]. The CS, BL, TY, 6-deoxoCS, TE, and 28-norCS are the most abundant BRs in plants [14,15,20]. The most extensive variety of BRs (2 conjugated and 25 free forms) was found in unripe bean (*Phaseolus vulgaris*) seeds [20]. Other legumes having fewer BR members have been found in shoots, seeds, and pollen, and their quantity was between 0.007 and 628l g/g fresh weight [14].

## 4. Chemical Structure of BRs

BRs have been categorized into three major types on the basis of each steroid molecule’s carbon number (i.e., C_27_, C_28_, and C_29_) [23]. The 5α-cholestane skeleton is the basic structure of C_27_-BRs, 5α-ergostane for C_28_-BRs, while 5α-stigmastane is the basic structure of C_29_-BRs. The structure of these hormones differs because of the type and orientation of oxygenated functions of A and B rings, and the number and position of functional groups in the side chain of the molecule. These changes occur as the result of oxidation and reduction reactions during biosynthesis. In general, BRs have been classified into conjugated (5) free, and (64) compounds [23].

BRs have vicinal hydroxyl groups in relation to the A-ring at C-2α and C-3α. BRs with α and β-hydroxyls, or ketone at the C-3 position are the progenitors of the BRs with 2α, 3α-vicinal hydroxyls. BR containing 2α, 3β-, 2β, 3α-, or 2β, 3β-vicinal hydroxyls maybe the precursors of 2α, 3α-vicinal hydroxyls. Two 2α, 3α-vicinal hydroxyl groups on the A-ring are responsible for overall structural attributes of the most active BRs, such as BL and CS. The declining order of activity 2α, 3α > 2α, 3β > 2β, 3α > 2β, 3β suggests that the α-oriented hydroxyl group at C-2 is essential for biological activity of BRs in plants [24,25]. On the basis of cholestane side chain, BRs have been divided by different substituents into C-23, C-24, and C-25: 23-oxo (4 compounds), 24-methylene (3 compounds), 24*S*-ethyl (4 compounds), 24*R*-methyl (5 compounds), 24*S*-methyl (23 compounds), C-25, 24-ethylidene (3 compounds), 24-methyl-25-methyl (3 compounds), 24-methylene-25-methyl (6 compounds) without substituent at C-23 (3 compounds), without substituent at C-24 (8 compounds), and without substituents at C-23, C-24 (2 compounds) [26,27,28].

BRs are classified into 6-oxo (6-ketone) (34 compounds), and 6-deoxo (non-oxidized) (21 compounds), and 7-oxalactone (12 compounds) types depending on B-ring oxidation stage. However, only one fourth type BR with hydroxyl group at C-6, such as 6α-hydroxycastasterone (6α-OH-CS) has been observed. In contrast, two compounds, for instance, 28-nor-(22*S*)-22-hydroxycampestrol (28-nor-22-OHCR) and (22*S*)-22-hydroxycampestrol (22-OHCR) were identified as a fifth type of BRs. Generally, 7-oxalactone BRs have been observed to show stronger biological activity compared to 6-deoxo type, and 6-oxo type. Sometimes 6-oxo BRs show activity similar to 7-oxalactone compounds, while non-oxidized BRs exhibit essentially little activity in the bean internode test or very little in the rice lamina inclination test [29,30,31].

## 5. Role of BRs in Growth and Development of Plants

**Figure 1:**
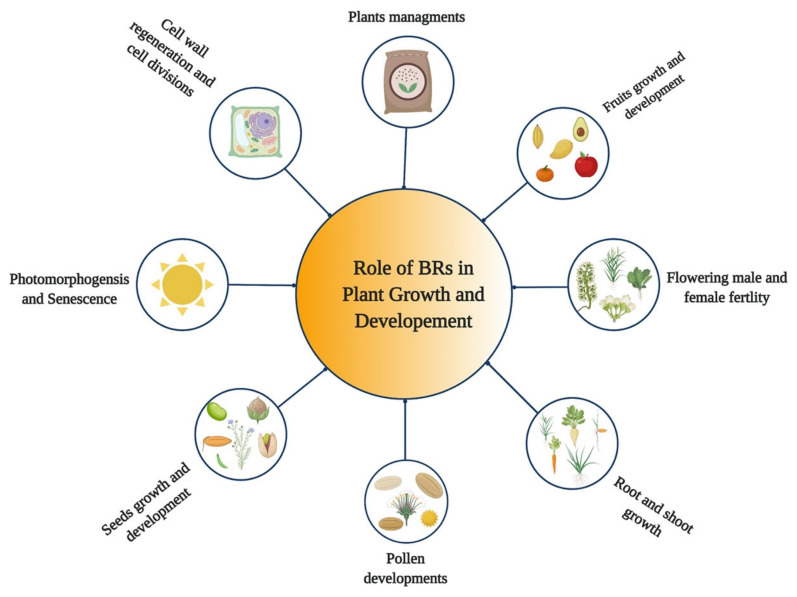
Role of Brassinosteroids (BRs) in growth and development of plants.

BRs are steroid hormones that play various roles in the growth and development of plants [32,33,34] (Figure 1). BRs regulate various developmental and physiological processes in plants, such as expansion, cell division, stem cell maintenance, vascular development, elongation of different cell types, and floral transition [35,36,37,38]. Moreover, they play diverse roles in hypocotyl elongation [39], root growth [7,40], shoot growth [28], stomata patterning [41,42], pollen tube growth, seed germination, and pollen germination and development [43], treachery element differentiation [44], xylem formation [45,46], xylem differentiation, photomorphogenesis and plant reproduction [47,48], and senescence [49]. BRs have the ability to activate the cell cycle during seed germination [50], regulate cell cycle progression [51], control leaf cells’ proliferation [52], and induce excessive growth in hydroponically grown plants [53]. BRs also regulate the abiotic and biotic stress responses and stomata development [7,34,54]. Moreover, BR plays a vital role in regulating male and female fertility in crops [55,56]. BR plays a role in etiolation and promotes the elongation of stigma [19], plant architecture, thermo-tolerance, proton transport, tiller number, leaf angle, and leaf size [57,58]. In addition, the exogenous application of BR or alteration in their biosynthesis and signaling could improve crop yields [59,60].

BRs are also involved in the regulation of several genes in plants (Table 1). In a study, Chen et al. [61] found that BRs induced *WRKY46*, *WRKY54*, and *WRKY70* genes that were observed to play positive roles in BR-regulated plant growth in *A. thaliana*. In another study, the histone lysine methyltransferase SDG8 is involved in BR-regulated gene expression. The knockout mutant *sdg8* displayed a reduced growth phenotype with compromised BR responses in *A. thaliana* [62]. In *A. thaliana*, BR regulates the seed development and affects the seed size/weight and number by transcriptionally modulating the genes and pathways that regulate the development of the seed and ovule [63,64]. Furthermore, BRs regulate root Nitrogen foraging response in *A. thaliana* during mild Nitrogen deficiency. A Brassinosteroid signaling kinase 3 (*BSK3*) gene is involved in the elongation of primary root during mild Nitrogen deficiency [65].

BR regulates the grain yield and plant architecture in rice [23]. The gene products of *BRD1* and *D11* are involved in the biosynthesis of BR, and affect the height of rice plants [66]. The *OsDwarf2/OsDwarf1* reduction encodes a C-6 oxidase needed for BR biosynthesis in rice, reducing second internode and seed length elongation [66,67]. In rice, BRs mediate the effects of N fertilization on spikelet development and contribute to promote spikelet growth by increasing the level of antioxidant system (AOS) and energy charge during panicle development [68]. In maize, inadequate BR biosynthesis causes male sterility due to failure of pollen and anther growth [55,56]. In cotton, both the fiber initiation and elongation of cultured cotton ovules have been reported to require BRs [69,70]. Moreover, an exogenous application of EBR delays the vegetative to generative transition in monocotyledonous wheat (*Triticum aestivum* L.). Brassinazole, a BR inhibitor, accelerates the transition and heading stage [71]. The application of BR accelerated winter rapeseed maturation by 4 to 8 days [72].

Priming of lucerne seeds with BL considerably increased length, vigor, and fresh and dry shoot and root weights [73]. The application of BR promoted the ripening of grape berry [74]. Treatment with EBR was observed to substantially increase sucrose synthase (sucrolytic) and soluble sugars content in berries [75]. Treatment with BR helped in reducing the decay of jujube fruits, likely due to its ability to postpone senescence and induce disease resistance [76]. Additionally, in potato tubers, the BRs have been shown to promote apical meristem growth [77], accelerating the cell division rate in isolated protoplasts of *Petunia hybrida* [78]. In *Pharbitis nil*, CS and BL treatments inhibit flowering in combination with the inductive photoperiod, implying that BR works in tandem with environmental cues to ensure the proper reproductive transition [19,79].

BRs can participate in physiological processes in response to stress by tuning plant growth, and improving plant performance by interacting with plant growth regulators or other plant hormones [80,81]. The disruption of BR signaling affects several developmental processes, including seed development [64], pollen development [82], and flowering time [83]. Plants with BR deficiency are dwarfed [84], and exhibit altered stomatal development [41,85], reduced male fertility, shortened hypocotyls, petioles and internodes, downward curled leaves, and delayed flowering [82]. Moreover, BR-deficient plants also have a compact plant structure because of the reduced lamina inclination. However, BR deficiency can reduce grain size, seed fertility and tiller number [9], improper stomatal distribution, and reduce seed germination [86]. BR-insensitive and -deficient mutants are often referred to as late flowering due to their retarded growth [87,88]. In contrast, plants over accumulating BRs display elongation of petioles and hypocotyls and increased height [89].

**Table 1:** Involvement of various Brassinosteroid--regulated genes in plant growth and development.

Gene	Description of Gene	Crop/Plant	Role in Growth	Reference
*CESA*	The *CESA* gene superfamily, encoding the catalytic subunits of cellulose synthase	Arabidopsis (*A. thaliana*)	Plays a role in regulating the cellulose synthesis	[90]
*CYCD3;1*	Cell division markers	Arabidopsis (*A. thaliana*)	Needed for normal cell cycle progression	[51]
Histone lysine methyltransferase *SDG8*	In Arabidopsis, there are 43 SET Domain Groups (*SDG*), which contain proteins with conserved SET domains	Arabidopsis (*A. thaliana*)	Involved in BR-regulated gene expression	[62]
*WRKY46*, *WRKY54*, and *WRKY70*	The *WRKY* family TFs are composed of over 70 members in Arabidopsis	Arabidopsis (*A. thaliana*)	Play positive roles in BR-regulated plant growth and drought stress	[61]
Brassinazole-resistant 1 (BZR1), and BES1-interacting MYC-like proteins (*BIMs*)	BZR1; BR-activated transcription factor (TF) and BIMs; bHLH TF	Arabidopsis (*A. thaliana*)	BR signaling promotes vegetative growth by inhibiting the floral transition	[91]
Transcripts of autophagy-related genes (*ATGs*)	Autophagy-related genes	Tomato (*Solanum lycopersicum*)	Enhanced level of BR triggers *ATGs* and formation of autophagosomes	[92]
*VvHMGR*	Plays a role in the mevalonate (MVA) pathway	Grape berries (*Vitis vinifera*)	Involved in increasing the anthocyanin content and promoting coloration. Accumulates the fruit sugar components, and decreases the tartaric acid content	[93]

## 6. Role of BRs against Different Stresses in Plants

**Figure 2:**
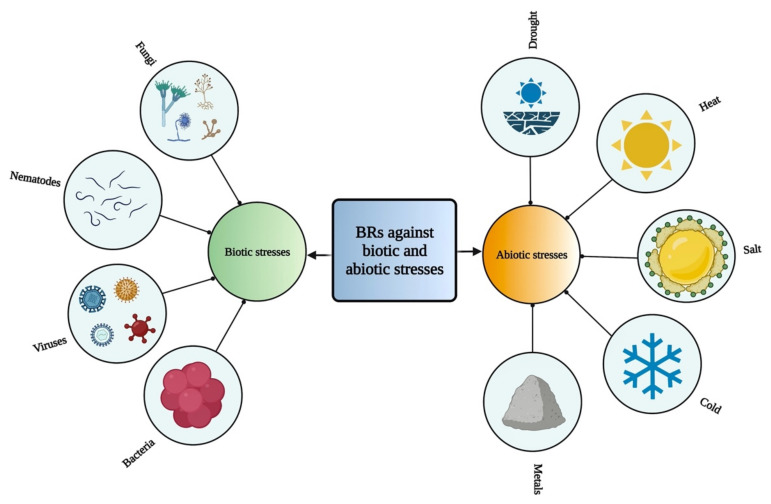
Role of BRs against different biotic and abiotic stresses in plants.

BRs play various roles against different kinds of biotic and abiotic stresses [94,95] (Figure 2). Various studies have shown that BRs play an essential role in acclimation to environmental stresses, resistance to pathogens, and cell elongation, resulting in increased crop yield and plant growth [13]. Therefore, these compounds can be used as biostimulants in crops to induce abiotic stress tolerance and to improve plant efficiency [80]. These compounds play a vital role to alleviate various stresses, such as drought [96,97], cold [98,99], heat [100,101], and salinity [102,103] by increasing the photosynthesis and biomass, strengthening antioxidant enzymes and the potential of detoxification as well as stimulates the expression of related genes [104,105]. BRs are also involved in oxidative stress, heavy metal stress response, and pathogen attack [104,106]. BRs play a crucial role in protecting plants from antimony (Sb) toxicity [107]. BRs have been involved in regulating various metabolic pathways and also interact with many other plant growth regulators [59]. However, these regulatory functions indicate the important roles of BR in adapting to environmental changes [108].

Several studies have reported that BRs regulate many genes against different stresses in various crops (Table 2). Earlier studies indicated that BRs play positive roles in drought tolerance in *Brassica napus*, *A. thaliana*, and wheat (*T. aestivum* L.) [109]. For example, overexpression of *A. thaliana* BR biosynthetic *AtDWARF4* gene in *B. napus* increased drought resistance [110]. In barley, the leaf disease at the tillering phase caused by *Helminthosporium teres* Sacc was reduced using 24 EBL [16]. The application of BRs has the potential in inducing tolerance against various plant diseases caused by the Tobacco mosaic virus (TMV) in tobacco and *Xanthomonas oryzae* and *Maganoprothe grisea* in rice [16,111]. The use of BRs has the potential to enhance defense against plant virus response by inducing several resistance genes and activating various vital antioxidant enzymes. Moreover, *Cucumber mosaic virus* (CMV) stress tolerance is stronger in bes1-D. However, BR signaling is required for BR-induced resistance to plant virus. In response to CMV infection, BR signaling can induce the expression of several resistance genes [112]. Furthermore, in response to chilling stress in tomato, the BRs regulate the NBR1-dependent selective autophagy in a BZR1-dependent manner [113]. The exogenous application of EBR and 28-homobrassinolide (HBL) has been shown to mitigate the harmful effects of heavy metals on plants [114,115]. Moreover, Exogenous application of BR was observed to increase pepper tolerance against low-temperature stress [116].

In a study, two important BR signaling components were shown to modulate the freezing tolerance in *A. thaliana*. The loss-of-function mutation in GSK3-like kinases (involved in BR signaling, bin2-3 bil1 bil2) mutants showed increased resistance to the freezing, while BIN2 overexpression exhibited hypersensitivity to freezing stress. By contrast, gain-of-function mutants of BZR1 and BES1 TFs showed increased resistance to the freezing [99]. The UBC32, a stress-induced functional ubiquitin conjugation enzyme (E2), which is localized in the ER membrane, connecting the ERAD process and BR-mediated growth promotion and tolerance to the salt stress. The mutant forms of BRI1, bri1-5, and bri1-9 were observed to be accumulated by the UBC32 mutation, and these mutant forms then activated the BR signal transduction [102]. *A. thaliana WRKY46*, *WRKY54*, and *WRKY70* TFs were shown to play roles in plant growth and drought response regulated by the BR—as the *wrky46 wrky54 wrky70* triple mutant exhibits defects in BR-regulated growth and more tolerance to the drought stress. *WRKY54* interacts directly with BES1 for co-regulating the expression of target genes [61]. Moreover, Eremina et al. [98] showed that BRs regulate the freezing tolerance. BR signaling-defective mutants were found to show hypersensitivity to freezing before and after the cold acclimation in *A. thaliana*. In contrast, the constitutive activation of BR signaling showed more resistance to freezing.

Another study was conducted in order to check the response of BR on cadmium’s effects on active oxygen metabolism and photosynthetic machinery in two tomato cultivars. The results showed a significant decrease in photosynthetic parameters, activity of several enzymes (carbonic anhydrase and nitrate reductase), and leaf water potential with the increasing levels of cadmium in the soil. BRs exogenous application increased the activity of photosynthetic machinery and antioxidant defense system, and nullified the detrimental effects of metal on these parameters [117]. A study in tomato shows the relationship between BR and ABA in inducing the production of H_2_O_2_ and their functions against paraquat (PQ) oxidative and heat stresses. Both BR and ABA induced increases in *RBOH1* gene expression levels, tolerance to the heat and PQ stresses, NADPH oxidase activity, and accumulation of apoplastic H_2_O_2_ in wildtype plants [118].

**Table 2:** Regulation of different stress-related genes by BRs.

Gene/BRs	Gene Function	Crop/Plant	Stress Type	Reference
Respiratory burst oxidase homolog (*RBOH*)	Involved in ROS generation	Cucumber (*Cucumis sativus* L.)	Cold and photo-oxidative stresses	[119]
*DREB*	Involved in regulating various cold stress-responsive genes	Rice (*O. sativa* L.)	Cold stress	[109,120]
Proline-5-caryboxylate synthetase 1 (*P5CS1*)	Involved in the proline biosynthesis	Arabidopsis (*A. thaliana*)	Salt stress	[121]
Abscisic acid stress ripening (*ASR*)	Involved in signal transduction	Mango (*Mangifera indica* L.)	Cold stress	[122]
YODA (*YDA*)	A TF involved in regulating stomatal conductance	Arabidopsis (*A. thaliana*)	Drought and salt stresses	[41]
*CYP90b3*, *GSH1*, and *GST1*	Play a role in detoxification	Tomato (*S. lycopersicum* L.)	Phenanthrene stress	[123]
Remorin	Membrane skeleton protein	Mango (*M. indica* L.)	Drought stress	[122]
*UBC32*	A stress-induced functional ubiquitin conjugation enzyme (E2)	Arabidopsis (*A. thaliana*)	Salt stress	[102]
Lipocalins	Involved in signal transduction	Mango (*M. indica* L.)	Cold stress	[122]
Submergence 1A (*SUB1A)*	An ethylene response factor (ERF), involved in conferring the submergence tolerance	Rice (*O. sativa* L.)	Submergence tolerance	[124]
Alternative oxidase (AOX)	Involved in protecting the plant photosystems	Tobacco (*Nicotiana benthamiana*)	Cold stress	[125]
Ferritin	Involved in iron storage	Rice (*O. sativa* L.)	Pesticide and salt stresses	[126]
Respiratory burst oxidase homolog 1 (*RBOH1*)	Involved in ROS generation	Tomato (*S. lycopersicum*)	Heat tolerance	[118]
Ascorbate peroxidase (*APX*)	Involved in the scavenging of ROS	Rice (*O. sativa* L.)	Pesticide and salt stresses	[127,128]
*bes1-D*	BRI1 EMS SUPRESSOR 1	Arabidopsis (*A. thaliana*)	Tolerance to *Cucumber mosaic virus* (CMV)	[112]
Superoxide dismutase (*SOD*)	H_2_O_2_ biosynthesis	Rice (*O. sativa* L.)	Pesticide and salt stresses	[127,128]
Glutathione reductase (*GR*)	Involved in the scavenging of ROS	Rice (*O. sativa* L.)	Pesticide and salt stresses	[127,128]
Catalase (*CAT*)	Engaged in the scavenging of ROS	Rice (*O. sativa* L.)	Pesticide and salt stresses	[127,128]
No-expressor of pathogenesis-related genes1-1 (*NPR1-1*)	Involved in regulating various stress-responsive genes	Arabidopsis (*A. thaliana*)	Salt and hyper-thermal stresses	[129]
1-aminocyclopropane-1-carboxylate synthase (*ACS*)	An ethylene synthesis enzyme	Tomato (*S. lycopersicum*)	Salt stress	[103]
Cesta (*CES*)	TFs that are involved in regulating several cold stress-responsive genes	Arabidopsis (*A. thaliana*)	Cold stress	[98]
*BZR1* and *BES1*	Basic helix-loop-helix TFs play a role in the BR-signaling pathway	Arabidopsis (*A. thaliana*)	Freezing tolerance	[99]
*WRKY*	Involved in regulating various stress-responsive genes	Arabidopsis (*A. thaliana*)	Drought stress	[61]
*BRL3*	A vascular-enriched member of the BR receptor family	Arabidopsis (*A. thaliana*)	Drought stress	[96]
*BZR1*	The main regulator of BR response	Tomato (*S. lycopersicum*) and Arabidopsis (*A. thaliana*)	Thermotolerance	[100,101]

## 7. BRs Signaling in Plants

**Figure 3:**
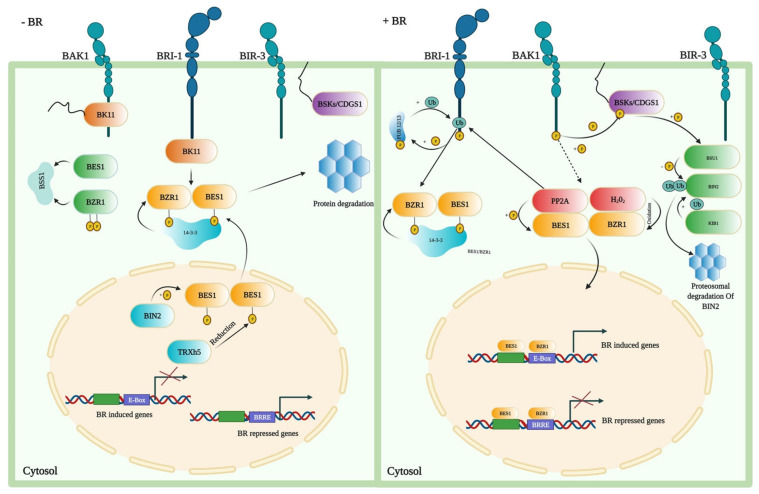
Signaling in the absence and presence of BRs in *A. thaliana*. When BRs are absent, BZR1 and BES1 proteins are being phosphorylated by the BIN2 that activates them by promoting binding of these proteins to the 14-3-3 proteins, resulting in cytoplasmic retention and degradation. This enhances the cytoplasmic retention of TFs, preventing them from entering the nucleus and terminating the response induced by the BR. When BRs are present, BR binding to BRI1 and the co-receptor BAK1 causes BKI1 to dissociate from BRI1 and causes trans-phosphorylation between BRI1 and BAK1. Through direct phosphorylation, the activated BRI1–BAK1 receptor complex transmits its signal to BSKs and Constitutive differential growth 1 (CDG1). BSU1 phosphatase is activated by BSKs or CDG1. BSU1 subsequently dephosphorylates the BIN2 to inactive it, and the E3 ligase KIB1 mediates the degradation of BIN2. Meanwhile, PP2A dephosphorylates BZR1 and BES1 to activate them, allowing TFs to enter the nucleus and regulate the expression of the BR target genes, either by direct interaction or through interactions with other TFs. Moreover, PP2A positively regulates BR signaling by the dephosphorylation of BZR1 and BES1, while the SBI1 (Suppressor of BRI1) deactivates the BRI1 through PP2A methylation.

In the last two decades, the BR signal transduction pathway has been extensively studied and reported as a complex pathway. The transduction pathway has a critical role in the growth and development of plants. The signal transduction pathway demonstrates that plant-specific leucine-rich repeat (LRR) receptor kinase located on the plasma membrane perceives BRs outside the cell. BRI1 interacts with BRI1-associated receptor kinase 1 (BAK1) and regulates the important positive regulators of the BR signaling, BZR1 and BES1 [130]. Increased BR levels result in dephosphorylation of BZR1, which facilitates the binding of dephosphorylated BZR1 (dBZR1) to conserved E-boxes (CANNTG) and/or BRRE elements (CGTGT/CG) in target BR-responsive genes’ promoters (Figure 3) [131,132].

BRI1 activates BZR1 and BES1 downstream TFs for inducing stress tolerance [19,47,133]. Upon BR perception, BR signals are relayed to BES1 and BZR1 via a signaling cascade, which eventually controls the transcription of genes regulated by the BRs [7,9,134,135]. BAK1, another LRR receptor kinase, interacts with BRI1 and acts as a co-receptor. The bioactive form of BR, brassinolide (BL), enhances the interaction of BRI1 and BAK1 [136]. BAK1 triggers the intracellular signaling pathways that include the protein phosphatase BSU1, the serine/threonine-protein kinase BSK1, protein phosphatase 2A (PP2A) phosphatases, the Glycogen synthase kinase 3 (GSK3)-like kinase Brassinosteroid-insensitive 2 (BIN2), and BZR1 family TFs [8,137]. Therefore, mutations in genes encoding the BR synthesis and signaling pathways’ main components cause limited plant yield and fertility, impaired growth and development of the organ, and severe dwarfism [7,35].

Plant hormones often regulate the expression of a downstream gene through TFs. BR regulates the development of plants via TFs that either repress or induce downstream genes [138]. BRs have received much research attention in the last two decades due to their crucial roles in plant development and crop yield enhancement. Consequently, the BR signaling cascade in plants is one of the well-studied signaling pathways [139]. Many TFs have been identified as being involved in downstream BR signaling pathways. In the BR signaling pathway, BES1 and BZR1 are considered to be essential TFs. BES1 has been found to be 88% identical with BZR1. It has similar protein domains: a nuclear localization signal (NLS) in the N terminal, a PEST domain in the C terminus, and a serine-rich domain in the center [49]. BES1/BZR1 also interacts with several TFs, including *HAT1*, *MYB30*, *BIM1*, and *MYBL2*, to induce or reduce the expression of downstream genes and incorporate the BR and other signaling pathways [138,140,141,142,143].

## 8. Concluding Remarks and Future Perspectives

Plants are mainly exposed to a number of biotic and abiotic stresses that negatively affect the plants and lead to the crop production loss. In a result, plants have adapted different mechanisms against these stresses, including the production of several phytohormones. BRs are the hormones that regulate numerous physiological and developmental processes. BRs play a crucial role in major plant antioxidant processes, including the regulation and increase in plant tolerance to various stresses. Over the last few decades, multiple studies on BRs have attracted the attention of plant scientists because of their involvement in various developmental and physiological processes in plants. In addition to their well-known functions in growth, they are now being discovered to play crucial roles in resistance to several biotic and abiotic stresses. BRs mediate these responses by regulating a wide range of genes. However, further research needs to be conducted to deeply understand the role of BRs in plant growth and development, and against various stresses in plants.

## Data Availability

Not applicable.
